# Use of the cardiac power index to predict fluid responsiveness in the prone position: a proof-of-concept study

**DOI:** 10.1016/j.bjane.2024.844545

**Published:** 2024-08-06

**Authors:** Ji Young Min, Joon Pyo Jeon, Mee Young Chung, Chang Jae Kim

**Affiliations:** The Catholic University of Korea, College of Medicine, Eunpyeong St. Mary's Hospital, Department of Anesthesiology and Pain Medicine, Seoul, Republic of Korea

**Keywords:** Cardiac output, Fluid therapy, Intraoperative monitoring, Prone position

## Abstract

**Background:**

The primary aim of this proof-of-concept study was to investigate whether the Cardiac Power Index (CPI) could be a novel alternative method to assess fluid responsiveness in the prone position.

**Methods:**

Patients undergoing scheduled elective lumbar spine surgery in the prone position under general anesthesia were enrolled in the criteria of patients aged 19–75 years with American Society of Anesthesiologists (ASA) physical status I–II. The hemodynamic variables were evaluated before and after changes in posture after administering a colloid bolus (5 mL.kg^−1^) in the prone position. Fluid responsiveness was defined as an increase in the Stroke Volume Index (SVI) ≥ 10%.

**Results:**

A total of 28 patients were enrolled. In responders, the CPI (median [1/4Q–3/4Q]) decreased to 0.34 [0.28–0.39] W.m^−2^ (*p* = 0.035) after the prone position. After following fluid loading, CPI increased to 0.48 [0.37–0.52] W.m^−2^ (*p* < 0.008), and decreased SVI (median [1/4Q–3/4Q]) after prone increased from 26.0 [24.5–28.0] mL.m^−2^ to 33.0 [31.0–37.5] mL.m^−2^ (*p* = 0.014). Among non-responders, CPI decreased to 0.43 [0.28–0.53] W.m^−2^ (*p* = 0.011), and SVI decreased to 29.0 [23.5–34.8] mL.m^−2^ (*p* < 0.009). CPI exhibited predictive capabilities for fluid responsiveness as a receiver operating characteristic curve of 0.78 [95% Confidence Interval, 0.60–0.95; *p* = 0.025].

**Conclusion:**

This study suggests the potential of CPI as an alternative method to existing preload indices in assessing fluid responsiveness in clinical scenarios, offering potential benefits for responders and non-responders.

## Introduction

Optimal fluid management guided by reliable hemodynamic indices is pivotal in resuscitation strategies.^1^^,^^2^ Traditional parameters like Central Venous Pressure (CVP) and Pulmonary Capillary Wedge Pressure (PCWP) have long been used to estimate intravascular volume and preload. Yet, their interpretation is complex due to influences from thoracic, pericardial, and abdominal pressures, vascular tone, myocardial function, and intrathoracic pressure.^3^ To mitigate these challenges, noninvasive dynamic preload indices such as Pulse Pressure Variation (PPV), Stroke Volume Variation (SVV), and Pulse Variability Index (PVI) have been proposed. These indices use heart-lung interactions to provide insights into fluid responsiveness, offering potential advantages over static measures.^4^ Despite their promise, the reliability of these dynamic indices can be compromised by alterations in intrathoracic pressure and tidal volume, which influence right ventricular stroke volume and intrathoracic blood volume during inspiration.^5^

The prone position, commonly utilized in spinal surgeries to optimize surgical access, significantly impacts blood pressure and cardiac function. This is primarily due to the compression of the inferior vena cava and increased intrathoracic pressure.^6^ Additional factors, such as pre-existing dehydration and challenges related to mechanical ventilation, further exacerbate hemodynamic instability in prone-positioned patients, potentially limiting the applicability of PPV, SVV, and PVI in guiding fluid management strategies. Recent studies have produced conflicting findings regarding the efficacy of goal-directed therapy based on indices such as PPV, SVV, and PVI in the prone position.^7^^,^^8^ These findings highlight ongoing debates and underscore the need for alternative methods to enhance the evidence-based efficacy of fluid management in these patients. Although another study has demonstrated that the Tidal Volume Challenge (TVC) test can partially address the limitations,^9^^,^^10^ the assessment process remains complex. It presents challenges when applied to patients with respiratory conditions. Consequently, novel alternative methods to assess fluid responsiveness in the prone position are required to guide fluid management in these complex clinical scenarios effectively.

The Cardiac Power Index (CPI) is a comprehensive marker for assessing cardiac function, as it integrates both the volumetric aspect (Cardiac Output, CO) and the pressure aspect (Mean Arterial Pressure, MAP) of cardiovascular performance. CPI is calculated using the formula:

$$CPI\;\left(W.m^{-2}\right)=CO\;\left(L.min^{-1}\right)*MAP\;\left(mmHg\right)*\;0.0022\;/\;Body\;surface\;area\;\left(m^{2}\right)$$

This offers a comprehensive measure of the heart's pumping capability and overall hemodynamic efficiency, reflecting both the volume of blood the heart pumps and the pressure it generates.^11^ The rationale for using CPI lies in its ability to indicate how efficiently the heart generates enough cardiac output relative to hemodynamic change.^12^ CPI assists clinicians in evaluating the heart's capacity to deliver sufficient blood flow across different hemodynamic conditions. In clinical settings, CPI has independently shown correlations with outcomes like mortality in patients with cardiogenic shock.^13^^,^^14^

Prone positioning can compromise the reliability of conventional fluid responsiveness indicators. The CPI, which offers a comprehensive assessment of both volumetric (CO) and pressure (MAP) aspects of cardiovascular performance, may be an alternative method in such scenarios. This prospective observational proof-of-concept study aims to objectively evaluate the efficacy of CPI in predicting fluid responsiveness during hemodynamic changes, particularly in response to postural shifts associated with the prone position.

## Methods

### Participant population

The study was conducted in a single-group prospective observational pilot study. This prospective observational pilot study, available on Research Square, was initially posted as a preprint (https://www.researchsquare.com/article/rs-2694372/v1) and did not undergo peer review at that stage. The present study was conducted at a single tertiary hospital (Eunpyeong St. Mary's Hospital, College of Medicine, The Catholic University of Korea) and adhered to the tenets of the Declaration of Helsinki and the principles of Good Clinical Practice. It was performed after obtaining approval from the Institutional Review Board (IRB) and Hospital Research Ethics Committee (The Catholic University of Korea, Eunpyeong St. Mary's Hospital IRB; IRB protocol nº PC22OISI0164; registered on 01/09/2022, President of the ethics committee: Jung Hwan Oh MD., Ph.D.). Registration to the Clinical Research Information Service (CRIS, https://cris.nih.go.kr, KCT0007800, accessed on 09/09/2022, with the first patient enrolled on 22/09/2022). This single-group study followed the relevant guidelines and regulations, and written informed consent was obtained from each patient before enrollment and after IRB approval.

### Inclusion criteria and exclusion criteria

Patients undergoing scheduled elective lumbar spine surgery in the prone position under general anesthesia were enrolled when they met the criteria of patients aged 19–75 years with American Society of Anesthesiologists (ASA) physical status I–II. Exclusion criteria encompassed cases of inaccurate arterial pressure waveforms (e.g., cerebrovascular disease, reduced ventricular function with left ventricular ejection fraction < 40%), preexisting severe valvular disease or cardiac arrhythmia, implanted pacemakers, unstable vital signs, and decreased renal function (serum creatinine > 2.5 mg.dL^−1^) due to reduced fluid excretion.

### Anesthesia and hemodynamic monitoring

Upon arrival in the operating room, patients underwent monitoring with 3-lead electrocardiography, peripheral oxygen saturation, and noninvasive arterial pressure. A rainbow sensor SET^TM^ (Masimo Corp., Irvine, CA, USA) was attached to each patient's index or middle finger to monitor PVI and Perfusion Index (Pi) continuously. The Bispectral Index (BIS) (Philips Medizin System Boeblingen GmbH Hewlett-Packard-Str. nº 271034; Boeblingen, Germany) was attached to the forehead, displaying the anesthesia depth. Anesthesia was induced with propofol bolus (1.5–2.5 mg.kg^−1^) and remifentanil (3.0 ng.mL^−1^) via Target-Controlled Infusion (TCI) using the Minto pharmacokinetic model. After confirming loss of consciousness, rocuronium (1.2 mg.kg^−1^) was administered for muscle relaxation. Intubation was performed, and mechanical ventilation was initiated with an air-oxygen mixture (fraction of inspired oxygen = 0.5) at an 8 mL.kg^−1^ tidal volume, calculated based on ideal body weight and an I:E ratio of 1:2 without Positive End-Expiratory Pressure (PEEP). The respiratory rate was adjusted to keep normocarbia. Anesthesia was maintained with sevoflurane (1.5–2.5%) and remifentanil infusion via TCI to achieve a BIS score of 40 to 60. After stabilizing mechanical ventilation and anesthesia, an A-line was placed on the radial artery opposite the finger with the rainbow sensor SET^TM^. Arterial pressure waveforms were monitored using the Pulsioflex system. (Pulsion Medical Systems AG, Munich, Germany). Five minutes after the commencement of A-line monitoring, hemodynamic and respiratory variables were recorded (T0). Before changing positions on the Jackson table, an additional 20 mg of esmeron was administered. Hemodynamic and respiratory variables were measured five minutes after prone positioning on the Jackson table (T1). Subsequently, 5 mL.kg^−1^ (ideal body weight) of 6% hydroxyethyl starch solution (HES 130/0.4; Volulyte, Fresenius Kabi, Stans, Switzerland) was administered over 10 minutes. Fluid loading was performed as determined by the attending anesthesiologist, and hemodynamic and respiratory variables were collected 5 minutes after fluid loading completion (T2). All measurements were recorded with no surgical procedures to eliminate confounding factors such as surgical stimulation, patient positioning, blood loss, or vasoactive drugs.

### Data collection

The hemodynamic variables including MAP, Pulse Pressure (PP), Heart Rate (HR), Stroke Volume Index (SVI), Cardiac Index (CI), PPV, SVV, PVI, CPI, and respiratory-related variables, such as Peak Inspiratory Pressure (PIP), dynamic lung Compliance (Cdyn), and dynamic Elastance (Edyn) were recorded. Cdyn and Edyn were calculated by a mechanical ventilator (MAQUET Flow I) using the following formula:

$$\begin{array}{c} \text{Cdyn}\left(L.cm^{-1}-1H_{2}O\right)=\text{Tidal}\;\text{volume}*\;\left(\text{Plateau}\;\text{airway}\;\text{pressure}-\text{PEEP}\right)-1\;\text{Edyn}\left(\text{cm}\;H_{2}O.L^{-1}\right)\\ =\text{Plateau}\;\text{airway}\;\text{pressure}*\text{Tidal}\;\text{volume}-1 \end{array}$$

Data collection occurred at three predefined time points: in the supine position (T0), 5 minutes after prone positioning on a Jackson table (T1), and 5 minutes after the completion of fluid loading (T2). Fluid responsiveness was determined based on the increase in SVI, classifying patients with an increase in SVI ≥ 10% as responders and those with an increase in SVI < 10% as non-responders.

### Primary outcome and secondary outcomes

The primary outcome of the present study focused on examining the correlation between CPI and fluid responsiveness in the prone position. The changes in other hemodynamic and respiratory-related variables were assessed as secondary outcomes.

### Sample size estimation

The statistical analysis involved recruiting 28 patients for the pilot study, considering a previously calculated Area Under the Receiver Operating Characteristic Curve (AUROC) of 0.969 for the predictive power of PPV in a previous study.^15^ The assumed predictive power for CPI was expected to be similar, and the number of participants was determined based on these assumptions, resulting in 24 calculated participants. The recruitment of 28 participants allowed for a 20% loss rate.

### Statistical analysis

R language version 3.3.3 and the T&F program ver. 3.0 were employed for statistical analyses. Missing data were excluded from the analysis. Various statistical tests were used for comparisons, such as the independent *t*-test, the Mann-Whitney *U* test for continuous variables, and the Chi-Square test or Fisher's exact test for categorical variables. A linear mixed model was used to analyze repeated measurements at multiple time points without corrections for multiple comparisons. Pearson's correlation evaluated the correlation between the change in SVI and hemodynamic variables after changing to the prone position. If statistically significant, logistic regression was performed to determine independent predictability for predicting responses. Cut-off values and AUROC curves for significant variables were constructed to predict fluid responsiveness. Data were presented as the number (percentage) and median [25^th^–75^th^ percentile, 1/4Q–3/4Q] for quantitative variables after checking for normality, and a significance level of *p* < 0.05 was applied.

### Gray zone approach

Data from multiple patients were collected to determine the gray zone, and CPI was recorded alongside fluid responsiveness measured by changes in Stroke Volume (SV). The relationship between these predictive variables and fluid responsiveness was analyzed by constructing an ROC curve and calculating the AUC to evaluate their accuracy. The optimal cutoff value was identified by balancing sensitivity and specificity as the ROC curve indicates, considering Youden's index for optimal threshold estimation. The gray zone is then defined as the uncertain range around this cutoff value (e.g., for PPV, between 9% and 13%), where the predictive variable's accuracy is not precise, requiring additional clinical judgment. Additionally, a second analytical approach defines three response classes: negative, inconclusive, and positive. The inconclusive gray zone is determined based on ranges with sensitivity below 88% and specificity below 85%.

## Results

### Participant demographics

The present study enrolled 28 participants. Of these, 15 (53.5%) were male, and 13 (36.5%) were female. The median age [1/4Q—3/4Q] was 66.5 [49.2–73.8] years in the responders and 65.5 [49.3–68.8] years in the non-responders. No significant differences in baseline characteristics were observed between the two groups. All enrolled patients maintained hemodynamic stability, and data collection was completed without adverse events. Basic demographic characteristics are presented in Table 1.

**Table 1:** Patient characteristics.

Characteristics	Subgroup	Responders (n = 8)	Non-responders (n = 20)	*p-*value
Age (years)		66.5 [49.3‒73.8]	65.5 [49.3‒68.8]	0.308
Height (m)		1.7 [1.6‒1.7]	1.6 [1.5‒1.7]	0.311
Weight (kg)		72.0 [59.9‒84.5]	60.0 [56.0‒64.0]	0.034^a^
IBW (kg)		61.5 [49.5‒67.0]	51.5 [47.0‒62.5]	0.318
BMI (kg.m^−2^)		26.8 [23.2‒30.3]	23.2 [21.65‒26.9]	0.134
Sex				0.686
	Male	5 [62.5]	10 [50]	
	Female	3 [37.5]	10 [50]	
ASA physical status				1.000
	I	1 [12.5]	4 [20]	
	II	7 [87.5]	16 [80]	
Comorbidities				0.432
	None	2 [25]	6 [30]	
	HTN	4 [50]	9 [45]	
	DM	0 [0]	1 [5]	
	HTN/DM	2 [25]	1 [5]	
Induction propofol dose (mg)		120.0 [102.8‒147.5]	105.0 [94.0‒110.0]	0.105
Total remifentanil dose (mg)		208.5 [191.3‒266.0]	192.5 [180.5‒225.8]	0.377
Fluid administration (mL)		307.5 [247.5‒335.0]	257.5 [235.0‒312.5]	0.318

Data are presented as median [1/4Q‒3/4Q], or numbers of patients (%).

Weight represents the absolute body weight.

IBW, Ideal Body Weight; BMI, Body Mass Index; ASA, American Society of Anesthesiologists; HTN, Hypertension; DM, Diabetes Mellitus.

a p < 0.05*.*

### Alterations in hemodynamic variables induced by changes in position

The changes in variables following the shift in position between the two groups are summarized in Table 2. In responders, the CPI (median [1/Q–3/4Q]) showed a significant decrease upon transitioning to the prone position, dropping from 0.48 [0.33–0.51] W.m^−2^ to 0.34 [0.28–0.39] W.m^−2^ (*p* = 0.035). Additionally, SVI (median [1/Q–3/4Q]) notably decreased from 29.0 [25.3–36.0] mL.m^−2^ to 26.0 [24.5–28.0] mL.m^−2^ (*p* = 0.042) in responders. In the prone position, responders demonstrated a significantly lower CPI of 0.34 [0.28–0.39] W.m^−2^ compared to non-responders with a CPI of 0.50 [0.34–0.60] W.m^−2^ (*p* = 0.002). Non-responders experienced no significant changes in CPI and SVI after transitioning to the prone position. There was no significant difference between PPV, SVV, and PVI in both groups due to postural changes. Both Cdyn and Edyns significantly decreased following the position change in both groups. No significant differences were observed in other measured hemodynamic variables between responders and non-responders in the prone position.

**Table 2:** Changes in hemodynamic variables before and after position change in responders and non-responders.

	Responders (n = 8)			Non-responders (n = 20)		
	Supine	Prone	*p*-value	Supine	Prone	*p-*value
**MAP (mmHg)**	83.0 [73.5‒92.8]	86.0 [79.5‒94.5]	0.183	84.5 [73.5‒91.8]	88.5 [81.0‒97.0]	0.135
**PP (mmHg)**	54.1 [45.0‒65.5]	49.8 [45.0‒54.0]	0.319	54.5 [ 43.0–64.0]	53.1 [40.0‒64.5]	0.674
**HR (beat.min^−1^)**	71.5 [60.8‒83.5]	66.0 [60.5‒77.3]	0.232	85.5 [69.5‒93.8]	77.5 [64.0‒94.3]	0.008^a^
**SVI (mL.m^−2^)**	29.0 [25.3‒36.0]	26.0 [24.5‒28.0]	0.042^a^	29.0 [25.3‒36.0]	32.0 [25.0‒35.5]	0.925
**CI (L.min^−1^.m^−2^)**	2.3 [2.0‒2.8]	1.9 [1.2‒2.2]	0.045^a^	2.2 [2.1‒2.3]	2.5 [1.9‒2.9]	0.260
**PPV**	11.6 [8.0‒14.5]	13.8 [9.0–17.5]	0.128	13.5[9.0 – 18.5]	14.3 [8.0‒16.5]	0.713
**SVV**	14.0 [12.0‒20.5]	18.0 [13.0‒24.3]	0.444	13.0 [12‒20.8]	13.5 [9.3‒20.0]	0.304
**PVI**	13.0 [6.0‒17.5]	13.5 [10.0‒20.0]	0.611	12.5 [6.5‒14.8]	9.0 [8.3‒16.0]	0.694
**CPI (W.m^−2^)**	0.48 [0.33‒0.51]	0.34 [0.28‒0.39]^b^	0.035^a^	0.45 [0.38‒0.60]	0.50 [0.34‒0.60]	0.823
**Ppeak (cm H_2_O)**	14.0 [12.25‒15.75]	16.5 [15.0‒18.5]	0.022^a^	13.0 [13.0‒14.0]	15.0 [14.0‒15.8]	<0.001^a^
**Cdyn (mL.cm^−1^ H_2_O)**	58.0 [45.25‒66]	45.0 [28.5‒53.5]	0.008^a^	55.0 [44.0‒65.0]	44.5 [34.8‒51.0]	<0.001^a^
**Edyn (cm H_2_O/l)**	18.0 [15.0‒20.0]	21.0 [18.0‒40.0]	0.036^a^	18.0 [15.0‒24.0]	23.0 [19.0‒29.0]	<0.001^a^

Data are expressed as median [1/4Q–3/4Q].

MAP, Mean Arterial Pressure; PP, Pulse Pressure; HR, Heart Rate; SVI, Stroke Volume Index; CI, Cardiac index; PPV*,* Pulse Pressure Variation; SVV, Stroke Volume Variation; PVI, Pleth Variability Index; CPI, Cardiac Power Index; Cdyn, Dynamic Compliance of Respiratory system; Edyn, Dynamic Elastance of the Respiratory System.

a p < 0.05 compared with the supine position in each group.

b p < 0.05 compared between responders and non-responders.

### Alterations in hemodynamic variables following fluid loading in the prone position

**Figure 1:**
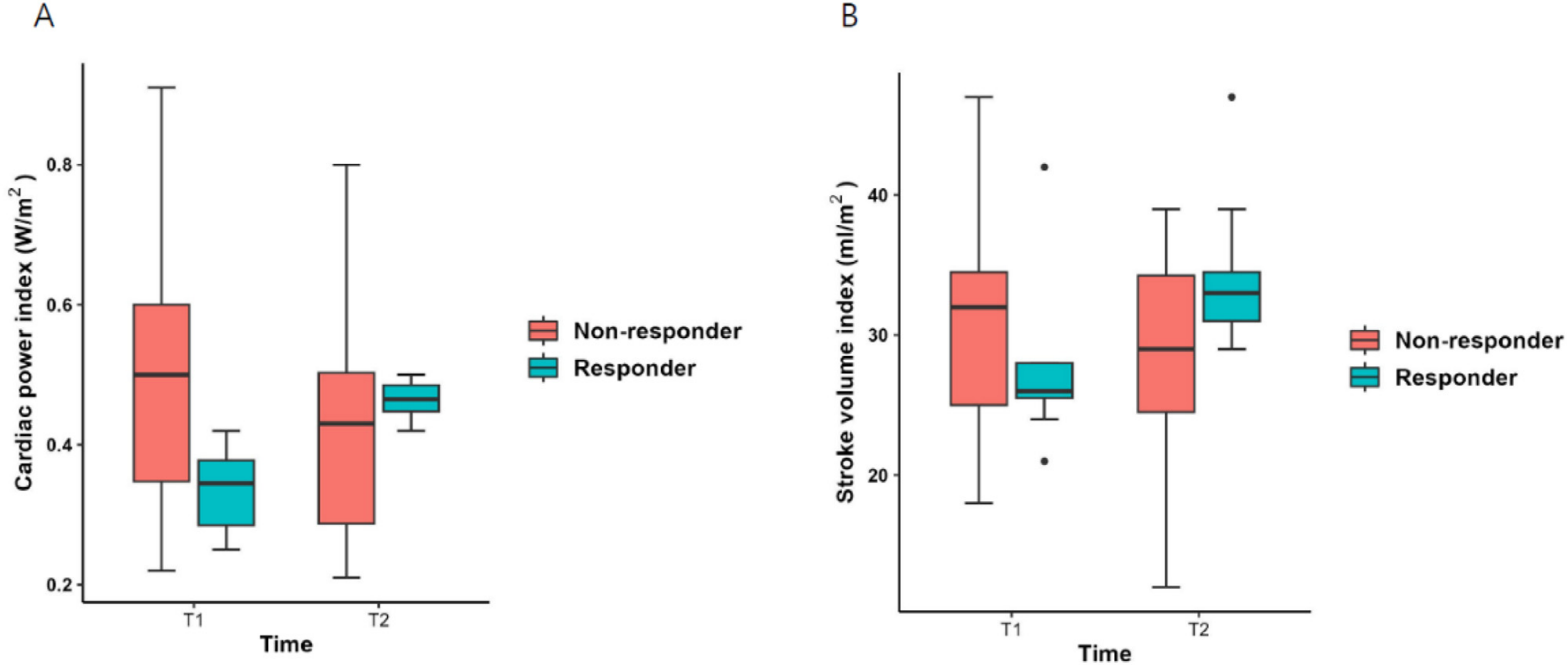
The numerical distribution of the cardiac power index (A) and stroke volume index (B) in the prone position before (T1) and after (T2) fluid loading is compared. The middle line represents the median, while the box indicates the Interquartile Range (IQR). This figure was created using the T&F program ver. 3.0 (YooJin BioSoft, Goyang, Korea).

Among the 28 enrolled participants, 8 (40%) responded to fluid loading (5 mL.kg^−1^ [ideal body weight]) in the prone position. Eight responders exhibited an increase in the CPI, with the SVI increasing by an average of 7.0%. In responders, the CPI exhibited statistically significant increases, rising from 0.34 [0.28–0.39] W.m^−2^ to 0.48 [0.37–0.52] W.m^−2^ (*p* = 0.008), and SVI increased from 26.0 [24.5–28.0] mL.m^−2^ to 33.0 [31.0–37.5] mL.m^−2^ (*p* = 0.014). The non-overlapping confidence interval (CI) for the initial and post-fluid loading SVI indicate that the change is statistically significant, suggesting the effect is genuine rather than due to random variation. Meanwhile, in non-responders, the CPI decreased from 0.50 [0.34–0.60] W.m^−2^ to 0.43 [0.28–0.53] W.m^−2^ (*p* = 0.011), and SVI decreased from 32.0 [25.0–35.5] mL.m^−2^ to 29.0 [23.5–34.8] mL.m^−2^ (*p* = 0.009) (Fig. 1). PPV, SVV, PVI, and HR significantly decreased in both groups after fluid loading (Table 3).

**Table 3:** Changes in hemodynamic variables before and after fluid loading in responders and non-responders in the prone position.

	Responders (n = 8)			Non-responders (n = 20)		
	Before	After	*p-*value	Before	After	*p-*value
**MAP (mmHg)**	86.0 [79.5‒94.5]	81.0 [72.5‒86.8]	0.439	88.5 [81.0‒97.0]	79.5 [72.5‒84.8]	0.042^a^
**PP (mmHg)**	49.8 [45.0–54.0]	46.2 [41.0–49.0]	0.253	53.1 [40.0‒64.5]	50.5 [34.5–56.5]	0.294
**HR (beat.min^−1^)**	66.0 [60.5‒77.3]	59.0 [53.8‒69.5]	0.016^a^	77.5 [64‒94.3]	67.0 [59.0‒76.5]	<0.001^a^
**SVI (mL.m^−2^)**	26.0 [24.5‒28.0]	33.0 [31.0‒37.5]	0.014^a^	32.0 [25.0‒35.5]	29.0 [23.5‒34.8]	0.009^a^
**CI (L.min^−1^.m^−2^)**	2.0 [1.7‒2.2]	2.2 [1.9‒2.4]	0.025^a^	2.5 [1.9–2.9]	2.3 [1.7‒2.6]	0.009^a^
**PPV**	13.8 [9.0‒17.3]	10.3 [5.5‒16.0]	0.014^a^	14.3[8.0‒16.2]	8.2 [6.0‒10.0]	0.005^a^
**SVV**	18.0 [13.0‒24.3]	11.0 [5.0‒14.8]	0.014^a^	13.5 [9.3‒20]	10.0 [7.3‒12.0]	0.002^a^
**PVI**	13.5 [10.0‒20.0]	6.5 [3.5‒13.3]	0.025^a^	9.0 [8.3–16.0]	8.0 [5.0‒12.0]	0.002^a^
**CPI (W.m^−2^)**	0.34 [0.28‒0.39]^b^	0.48 [0.37‒0.52]	0.008^a^	0.50 [0.34‒0.60]	0.43 [0.28‒0.53]	0.011^a^

Data are expressed as median [1/4Q‒3/4Q].

MAP, Mean Arterial Pressure; PP, Pulse Pressure; HR, Heart Rate; SVI, Stroke Volume Index; CI, Cardiac Index; PPV, Pulse Pressure Variation; SVV, Stroke Volume Variation; PVI, Pleth Variability Index; CPI, Cardiac Power Index

a p < 0.05 compared with before fluid loading in each group.

b p < 0.05 compared between responders and non-responders.

### Prediction of fluid responsiveness

**Figure 2:**
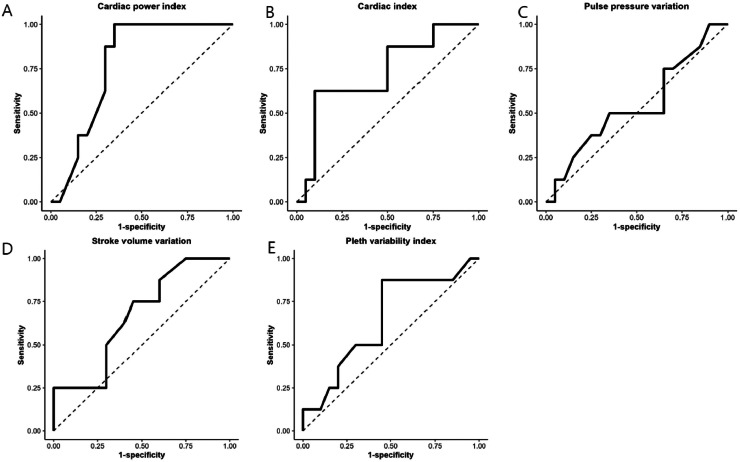
The Areas Under the Receiver Operating Characteristic (AUROC) curves of the Cardiac Power Index (A), Cardiac Index (B), Pulse Pressure Variation (C), Stroke Volume Variation (D), and Pleth Variability Index (E) for predicting fluid responsiveness are illustrated. In the prone position, the AUROC of the Cardiac Power Index was 0.78 (95% CI 0.60–0.95) with a cut-off value of 0.42 W.m^−2^ (sensitivity 100% and specificity 65%). This figure was created using T&F program ver. 3.0 (YooJin BioSoft, Goyang, Korea).

Pearson's correlation analysis demonstrated a direct relationship between changes in SVI and hemodynamic variables in the prone position (Table 4). The AUROC for predicting fluid responsiveness using prone CPI was 0.78 (95% Confidence interval [95% CI 0.60–0.95]; *p* = 0.025) with 100% sensitivity and 65% specificity (Table 4). The optimal CPI cut-off value in the prone position was ≤ 0.42 W.m^−2^ (Fig. 2A). AUROC for predicting fluid responsiveness using CI was 0.73 (95% CI 0.50–0.94), achieving a sensitivity of 63% and specificity of 90% (Fig. 2B). PPV and SVV had AUROCs of 0.54 (95% CI 0.35–0.73) with 50% sensitivity and 65% specificity and 0.67 (95%CI 0.47–0.83) with 75% sensitivity and 55% specificity, respectively (Fig. 2C and 2D). PVI had an AUROC of 0.65 (95% CI 0.45–0.82) with 88% sensitivity and 55% specificity (Fig. 2E).

**Table 4:** Comparison of area under the receiver operating characteristic curves of Cardiac Power Index (CPI) and dynamic preload index in the fluid responsiveness.

	Area under the curve	95 % CI	Sensitivity (95% CI)	Specificity (95% CI)	Cut-off	*p-*value
**CPI (W.m^−2^)**	0.78	0.60‒0.95	100 (1.00‒1.00)	65 (0.44‒0.86)	0.42	0.025^a^
**CI (L.min^−1^.m^−2^)**	0.73	0.50‒0.94	63 (0.29‒0.96)	90 (0.77‒1.00)	1.80	0.067
**PPV**	0.54	0.35‒0.73	50 (0.15‒0.85)	65 (0.44‒0.86)	14.5	0.722
**SVV**	0.67	0.47‒0.83	75 (0.45‒1.00)	55 (0.33‒0.77)	15.0	0.170
**PVI**	0.65	0.45‒0.82	88 (0.65‒1.00)	55 (0.33‒0.77)	9.50	0.232
**HR (beat.min^−1^)**	0.64	0.44‒0.81	75 (0.35‒0.97)	65 (0.40‒0.85)	69.0	0.234

CI, Confidence Interval; CPI, Cardiac Power Index; CI, CardiacI; PPV, Pulse Pressure Variation; SVV, Stroke Volume Variation; PVI, Pleth Variability Index; HR, Heart Rate.

a p < 0.05.

### The gray zone approaches

**Figure 3:**
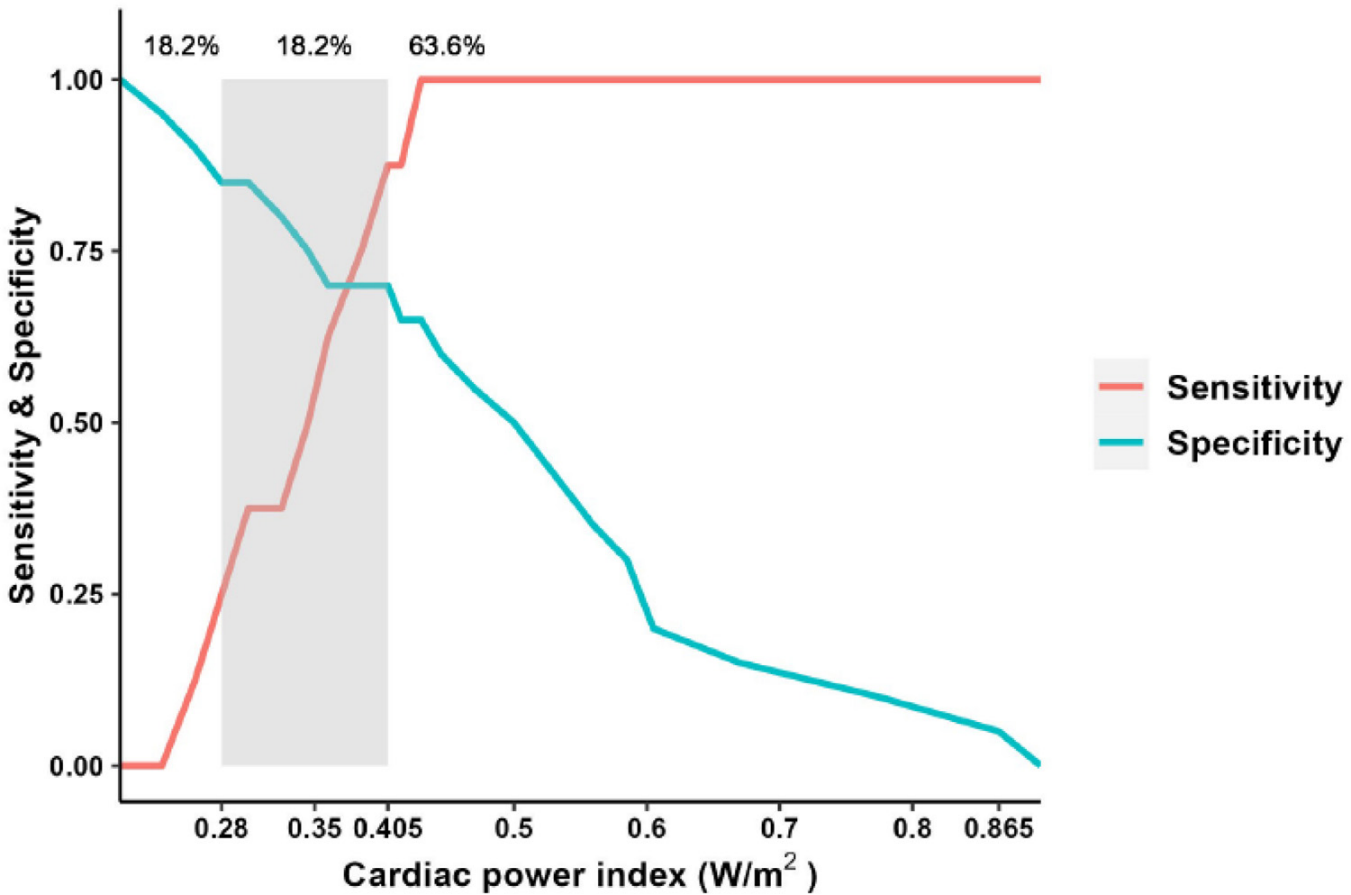
Gray zone approach. The figure illustrates the sensitivity and specificity associated with fluid responsiveness prediction on the left and right Y-axes, respectively, using different Cardiac Power Index (CPI) values as cut-offs along the X-axis.

ROC curve analysis using CPI for 1000 samples generated by 1000 random restorations using bootstrapping was performed to assess gray zones. The cut-off indicates the time to maximize Youden's J statistics (Table 5). Of the 28 patients in the present study, 63.6% (14 subjects) of samples had a sensitivity of 0.88 or higher (Areas with a CPI ≥ 0.41). Samples with a specificity of 0.85 or higher (Areas with a CPI ≤ 0.28) accounted for 18.2% (4 patients), and 18.2% (4 patients) belonged to the gray zone outside of the above area (Fig. 3). Patients in the 0.28 < CPI < 0.41 regions are ambiguous regarding the response prediction to fluid loading as a gray zone.

**Table 5:** Distribution of cardiac power index cutoff values using bootstrapping with iteration = 1000.

Average	Standard deviation	Lower 95% CI	Upper 95% CI
0.420	0.025	0.371	0.470

## Discussion

This proof-of-concept study represents the first exploration into the usefulness of CPI as an alternative method of assessing fluid responsiveness in the prone position for lumbar spine surgery patients. Positive responders to fluid responsiveness in the prone position exhibit a decreased CPI, suggesting a potential threshold (≤ 0.42 W.m^−2^) for predicting fluid responsiveness.

The prone position decreases SV and can lead to hypotension by elevating intrathoracic pressure, which hinders venous return.^16^ However, in the present study, the PIP remained below 20 cmH_2_O. Considering that a driving airway pressure of 20 cm H_2_O is recognized as the threshold significantly affecting the transmission of airway pressure to the intracardiac cavity,^15^ it is plausible that the PIP value in the present study might not have been high enough to impede venous return significantly. Despite a significant reduction in Cdyn of the respiratory system after prone positioning in the present study, it still exceeded the values reported in a previous study.^17^ Despite no definitive factor significantly reducing venous return, responders exhibited decreased SVI after position change, even with insignificant changes in venous return. Furthermore, the present study found a poor relationship between dynamic preload indices such as PPV, SVV, and PVI measured before prone positioning and changes in SVI and CI after the position change. These findings support the hypothesis that a decrease in SVI after a position change may be another plausible explanation.

One possible reason for the decreased SVI in responders is the impact of transitioning to the prone position on systolic performance. This interpretation is based on the close association of CPI with Ejection Fraction (EF)^18^ and its relationship with external cardiac work, reflecting both left ventricular systolic and diastolic performance.^11^ A previous study has demonstrated that body position changes can significantly alter cardiac function, such as decreased left ventricular EF and left atrium diameter in the prone position.^19^ The Jackson spine table used in the present study had a minor effect on intra-abdominal pressure by allowing it to be suspended freely and solely on cardiac function.^20^ However, the lumbar spine lordosis will inevitably decrease to have better access. Therefore, hemodynamic circulation is interfered with when we attempt to change the curvature of the spine with positioning devices. Thoracic compression may still persist.^21^ In the present study, Edyn was significantly decreased in all groups, indicating increased chest wall stiffness. Additionally, a decrease in preload, which conventional indices may not detect, could explain the decreased SVI. Research has shown a correlation between CPI and the Right Ventricular End-Diastolic Volume Index (RVEDVI),^22^ suggesting that a decrease in RVEDVI could lead to a decreased SVI, as reflected by a decrease in CPI. Therefore, the observed reduction in SVI in responders likely results from impaired systolic performance due to thoracic compression and undetected decreases in preload, as all reflected by a decrease in CPI.

After fluid loading, responders’ improvement in SVI can be attributed to the Frank-Starling mechanism,^23^ where the cardiac performance responds to increased preload (end-diastolic volume) by contracting more forcefully. The expanded Frank-Starling curve in responders suggests that their SVI increased through this physiological response, as evidenced by the elevated CPI. In contrast, non-responders displayed reduced dynamic preload indices and CPI after fluid loading, along with a decreased SVI. Despite the increased preload, non-responders did not effectively adjust their operating point on the pressure-volume diagram. This means that their cardiac performance did not contract more effectively despite the additional preload, leading to decreased SV. This inadequate response can be detrimental, potentially resulting in fluid overload and adverse effects. The key takeaway is that relying solely on dynamic preload indices to guide fluid therapy can be risky, particularly for certain patient groups experiencing hemodynamic instability. While dynamic preload indices are useful, they do not provide a complete picture of the heart's ability to handle increased preload. Given these considerations, exploring alternative strategies beyond fluid infusion is crucial. These alternatives might include using CPI to optimize treatment and improve patient outcomes during episodes of hemodynamic instability.

The correlation of CPI with cardiac performance in response to hemodynamic changes, such as fluid loading, provides alternative insights into the patient's cardiovascular status before intervention. This enables clinicians to predict the potential benefits and risks of fluid administration. Essentially, CPI helps assess the likelihood that administering fluids may lead to unfavorable outcomes, empowering clinicians to make more informed decisions regarding fluid therapy. As a result, it offers an additional approach for clinicians to understand hemodynamic changes and optimize fluid therapy tailored to individual patient needs. Moreover, the current study demonstrates that CPI moderately assesses diagnostic accuracy, with an AUROC value of 0.78 (95% CI 0.60–0.95). An AUROC value above 0.7 typically indicates diagnostic validity. The reported sensitivity of CPI is 100%, indicating its high capability in correctly identifying the condition. However, the specificity is 65%, suggesting a lower ability to avoid false positives. This implies that while CPI is highly effective in detecting the condition, it may also result in a notable rate of false positives. Clinicians should consider this limitation when interpreting results, as high sensitivity might lead to potential over-diagnosis in clinical practice. Therefore, while CPI shows promise as an alternative method for perioperative fluid management, its use should complement other clinical assessments.

The present study has several strengths in utilizing the CPI to assess fluid responsiveness. However, certain limitations must be acknowledged. Firstly, the fluid challenge test was not conducted with patients in the supine position. Conducting the test in this position would have allowed for comparing the effectiveness of CPI and dynamic preload indices in a more standardized position. Additionally, CPI should have been compared to dynamic tests such as the tidal Volume (Vt) challenge^24^ or lung recruitment maneuver^25^ during fluid loading. Such comparisons are crucial to establish the practicality and reliability of CPI in different clinical scenarios. Secondly, the present study involved spinal surgery using various prone positioning systems, including different frames like Wilson's and Andrew's, which may have distinct hemodynamic and respiratory effects. The present study's findings might have differed if a single type of frame, such as Wilson's or Andrew's, had been used.^26^ The variability in positioning systems introduces a potential confounder in interpreting the results. Thirdly, the fluid bolus was assessed after 5 minutes, which might introduce limitations due to the body's compensatory mechanisms. Ideally, the evaluation should be conducted within 2 minutes to capture the immediate hemodynamic response before the body begins to equilibrate.^27^ The timing of the assessment should be considered a limitation, and future protocols might benefit from earlier evaluation to improve accuracy and reliability. Finally, non-calibrated monitoring estimates CPI using indirect parameters, which can lead to limited accuracy due to the lack of personalized calibration.^28^, ^29^, ^30^ This method struggles to account for individual physiological differences, resulting in lower precision than calibrated techniques. These limitations are particularly critical in clinical situations where precise cardiac output estimation is essential.

## Conclusion

The findings from this proof-of-concept study, conducted on healthy patients with relatively normal heart function in the prone position, demonstrate that 8 out of 28 patients responded positively to fluid loading, showing an association between increased SVI and CPI. These results suggest that CPI may be an alternative method for assessing fluid responsiveness in patients in the prone position for lumbar spine surgery. Further research with a more extensive and diverse patient population is warranted to evaluate its clinical utility.

### Trial registration

KCT0007800 (accessed on 09/09/2022, with the first patient enrolled on 22/09/2022) https://cris.nih.go.kr/cris/search/detailSearch.do?search_lang=E&focus=reset_12&search_page=M&pageSize=10&page=undefined&seq=26647&status=6&seq_group=22909.

## Declaration of competing interest

The authors declare no conflicts of interest.
